# Isolation and characterization of a *Bacillus amyloliquefaciens* strain with zearalenone removal ability and its probiotic potential

**DOI:** 10.1371/journal.pone.0182220

**Published:** 2017-08-01

**Authors:** An Lee, Kuan-Chen Cheng, Je-Ruei Liu

**Affiliations:** 1 Graduate Institute of Food Science and Technology, National Taiwan University, Taipei, Taiwan; 2 Institute of Biotechnology, National Taiwan University, Taipei, Taiwan; 3 Department of Animal Science and Technology, National Taiwan University, Taipei, Taiwan; 4 Agricultural Biotechnology Research Center, Academia Sinica, Taipei, Taiwan; Georg-August-Universitat Gottingen, GERMANY

## Abstract

Zearalenone (ZEN) is a non-steroidal estrogenic mycotoxin produced by *Fusarium* species, which has been shown to be associated with reproductive disorders in livestock, and to a lesser extent with hyperoestrogenic syndromes in humans. The aim of this study was to characterize a *Bacillus amyloliquefaciens* strain with ZEN removal ability. A pure culture of a strain designated LN isolated from moldy corn samples showed a high ZEN removal capability. Based on microscopic observations, biochemical characteristics, and phylogenetic analysis of the 16S rRNA gene sequence, LN was identified as *B*. *amyloliquefaciens*. After incubation of *B*. *amyloliquefaciens* LN in Luria-Bertani (LB) medium containing 3.5 ppm of ZEN, the ZEN concentration fell below the detection limit within 24 h. In ZEN-contaminated corn meal medium, *B*. *amyloliquefaciens* LN decreased ZEN concentration by 92% after 36 h of incubation. In phosphate-buffered saline (PBS) containing 5 ppm of ZEN, *B*. *amyloliquefaciens* LN reduced the ZEN concentration from 5 ppm to 3.28 ppm immediately after coming into contact with ZEN, and further reduced the ZEN concentration to 0.36 ppm after 4 h of incubation. The amounts of ZEN adsorbed by the cells of *B*. *amyloliquefaciens* LN did not increase with the extension of incubation time, indicating that *B*. *amyloliquefaciens* LN not only possessed ZEN adsorption ability, but also exhibited the ability to degrade ZEN. In addition, *B*. *amyloliquefaciens* LN was non-hemolytic, non-enterotoxin producing, and displayed probiotic characteristics including acidic tolerance, bile salt tolerance, and anti-pathogenic activities. These findings suggest that *B*. *amyloliquefaciens* LN has a potential to be used as a feed additive to reduce the concentrations of ZEN in feedstuffs.

## Introduction

During the harvest, processing, transportation, and storage period, feeding stuffs and food ingredients may be contaminated by molds which produce toxic secondary metabolites, named mycotoxins. Mycotoxins are capable of causing diseases in humans and animals, and also cause economic loss due to the decreased quality and safety of crops, which ultimately can impact crop trade. Thus, mycotoxins can cause public health issues, economic loss, and food problems [1]. The major mycotoxin-producing molds include *Aspergillus*, *Claviceps*, *Fusarium*, and *Penicillium*, and the major mycotoxins produced include aflatoxins, deoxynivalenol, fumonisins, ochratoxin, and zearalenone (ZEN). Among these mycotoxins, ZEN is one of the greatest contributors to economic loss in swine production. ZEN is produced by *Fusarium* species, and the main species of ZEN-producing fungi are *F*. *graminearum* and *F*. *culmorum* [2–4]. Feedstuffs which easily contaminated by ZEN include corn, wheat, rice, barley, millet, and oats. A survey on the occurrence of ZEN in feedstuffs showed that ZEN was detected with an average level of 104 ppb with 45% contamination rate from 5,402 samples in Australia, 87 ppb with a 40% contamination rate from 1,402 samples in Japan, and 120 ppb with a 49% contamination rate from 1,820 samples in China [5]. ZEN possesses a phenolic ring; thus, ZEN may bind to cytosolic estrogen receptors present in the uterus and mammary glands, resulting in the reproductive disorders in animals, and also affecting the reproductive efficiency of swine [1]. A previous study reported that the feeding stuffs containing 1 ppm ZEN caused the reduction of crude protein digestibility and feeding efficiency in swine [6]; and the feedstuffs containing more than 1.1 ppm of ZEN damaged the uterus, ovaries, liver, kidney, spleen, and other organs in swine [7]. ZEN causes abnormal spindle fibers during cell division, resulting in infertility and abnormal ploidy embryos in pigs that consume ZEN for a long period of time [8]. Children who consume ZEN for a long period of time exhibit early sexual maturation, and in boys breast growth is observed [9]. Furthermore, ZEN also promotes the growth of human breast cancer cells [10].

Biological methods are preferred for removing mycotoxins in feedstuffs as they have the advantages of high specificity, require only mild conditions, and destroying minimal nutrient content [11]. There are two biological strategies for removing ZEN from feedstuffs and food ingredients. The first is to use biological mycotoxin-adsorbing agents, such as yeast cell walls derived from *Saccharomyces cerevisiae*, and some lactic acid bacterial strains such as *Lactobacillus rhamnosus* GG and *L*. *rhamnosus* LC-705 [12,13], to form a complex with ZEN, which then passes through the animal gastrointestinal tract and is eliminated via the feces. The second method is to use biotransforming agents, such as bacteria, yeast, fungi, and enzymes, to convert ZEN into less- or non-toxic metabolites [14–21].

*Bacillus amyloliquefaciens* is broadly used industrially to produce amylase and protease [22]. Recently, some *B*. *amyloliquefaciens* strains were reported to have probiotic potentials. For example, Islam et al. isolated a *B*. *amyloliquefaciens* strain from soil, and demonstrated it had a beneficial effect on inflammatory bowel disease [23]. Larsen et al. reported a *B*. *amyloliquefaciens* strain exhibited potential as a probiotic additives in pig feed [24]. Ahmed et al. reported a *B*. *amyloliquefaciens* strain had the potential to be used as a probiotic additive for broiler feed for improving growth performance [25]. Li et al found that broiler feed supplemented with *B*. *amyloliquefaciens* could partially alleviate the compromised growth performance and diminished immune system performance induced by lipopolysaccharides [26]. In addition, previous studies showed that some *B*. *amyloliquefaciens* strains possess aflatoxins, ochratoxin, or ZEN degrading ability [27–29]. For example, Siahmoshteh et al. reported a *B*. *amyloliquefaciens* strain could degrade aflatoxin B1 [27]. Chang et al. found a *B*. *amyloliquefaciens* strain displayed ochratoxin degrading activity [28]. Xu et al. reported a *B*. *amyloliquefaciens* strain showed an efficient ZEN degrading activity [29].

In the present study, we identified and characterized a *B*. *amyloliquefaciens* strain with ZEN removal properties. The probiotic properties, including acid and bile salt tolerance, adherence capability, and anti-pathogenic activities of this *B*. *amyloliquefaciens* strain were evaluated.

## Materials and methods

### Chemicals, reagents, and bacterial strains

ZEN was purchased from Sigma-Aldrich Co. (St. Louis, MO), and was dissolved in acetonitrile (0.5 mg mL^-1^) to prepare a stock solution. The solution was stored in the dark at -20°C, and brought to room temperature before use. The ZEN standard solutions for high-performance liquid chromatography (HPLC) calibration or spiking purposes were prepared daily by diluting the stock solution in methanol. Acetonitrile and methanol (HPLC grade) was supplied by J. T. Baker Inc. (Phillipsburg, NJ). Water for the HPLC mobile phase was purified successively by reverse osmosis and a Milli-Q system (Millipore, Bedford, MA). All the other chemicals used were of analytical reagent grade and obtained from Sigma-Aldrich. All solutions prepared for HPLC were filtered through a 0.22 μm-pore size nylon filter (Millipore) before use. All microbial strains used in this study are shown in Table 1.

**Table 1:** Microbial strains used in this study.

Strains	Relevant features	Reference or source
*Bacillus amyloliquefaciens* LN	The zearalenone-degrading strain isolated from moldy corn samples	This study
*Bacillus amyloliquefaciens* ATCC 23350	The type strain of *B*. *amyloliquefaciens*	American Type Culture Collection (ATCC; Manassas, VA, USA)
*Bacillus cereus* ATCC 11778	The *B*. *cereus* strain that produces Nhe enterotoxin	ATCC
*Bacillus cereus* ATCC 33019	The *B*. *cereus* strain that produces Hbl and Nhe enterotoxins	ATCC
*Escherichia coli* O157:H7 ATCC 35150	The enterohemorrhagic *E*. *coli* strain of O157:H7 serotype	ATCC
*Fusarium graminearum* ATCC 26557	The *F*. *graminearum* strain that produces zearalenone	ATCC
*Listeria monocytogenes* BCRC 14930	The *L*. *monocytogenes* strain isolated from food sources	Bioresource Collection and Research Center (BCRC; Hsinchu, Taiwan)
*Listeria monocytogenes* BCRC 15338	The *L*. *monocytogenes* strain isolated from spinal fluid of child with meningitis	BCRC
*Listeria monocytogenes* BCRC 15387	The *L*. *monocytogenes* strain isolated from cerebrosinal fluid of child with meningitis	BCRC
*Salmonella enterica* subsp. *enterica* BCRC 12947	The type strain of *Salmonella typhimurium*	BCRC

### Isolation of the bacterial strains with ZEN removal ability

A total of 148 bacterial strains were isolated from the moldy corn samples that were obtained from the Experimental Farm of National Taiwan University (Taipei, Taiwan), according to the methods described by Petchkongkaew et al. [30]. A single colony of each bacterial strain was transferred to Luria-Bertani (LB) broth (Difco Laboratories, Detroit, MI) containing 3.5 ppm ZEN, and incubated at 37°C in an orbital shaker (Major Science Inc., Taipei, Taiwan) at 250 rpm for 24 h. Then, the bacterial culture was centrifuged at 5,000*×g* for 20 min at 4°C (Tomy MX-301, Tomy Digital Biology Co., LTD, Tokyo, Japan), and the supernatant was collected. ZEN concentration was quantified using an AgraQuant ZON test kit (Romer Labs Inc., Union, MO). A pure culture of the bacterial isolates that showed highest ZEN removal ability was designated as strain LN, and was subjected to further analysis. The bacterial strain LN has been deposited in the German Collection of Microorganisms and Cell Cultures GmbH (Deutsche Sammlung von Mikroorganismen und Zellkulturen GmbH; DSMZ), with deposit number DSM 32119.

### Phenotypic characteristics of the strain LN

In order to observe colony morphology, the strain LN was cultivated on LB agar plate or blood agar plates (Merck, Darmstadt, Germany) at 37°C for 24 h and then observed. For morphological observation of the cells, the strain LN was grown in LB broth and incubated at 37°C in an orbital shaker at 250 rpm for 16 h. The cells were harvested by centrifugation at 5,000×*g* for 20 min at 4°C. The cells were stained with 4’,6’-diamidino-2-phenylindole (DAPI) according to the method described by Waldeck et al. [31], and observed under a fluorescence microscope. Cells were also stained with a Gram Staining kit (Sigma-Aldrich Co., USA) according to the manufacturer’s instructions, and then observed under a microscope. Unstained cells were observed under a phase-contrast microscope.

### Biochemical characteristics of the strain LN

To determine biochemical characteristics, the carbon source utilization profiles of the strain LN was determined using an API 50 CHB system (bioMerieux, Inc., Marcy l'Etoile, France) according to the manufacturer’s instructions. The *B*. *amyloliquefaciens* type strain ATCC 23350, obtained from the American Type Culture Collection (ATCC; Manassas, VA), was used as a reference strain.

### Molecular identification of the strain LN

Molecular identification of the strain LN was performed by the methods described by Weisburg et al. [32]. Genomic DNA of the strain LN was isolated using the DNeasy Blood & Tissue kit (Qiagen Inc., Valencia, CA). The standard 16S rRNA gene primers, 16S-27f (5’ AGAGTTTGATCMTGGCTCAG 3’) and 16S-1492r (5’ CGGTTACCTTGTTACGACTT 3’), were used for polymerase chain reaction (PCR) to amplify the partial 16S rRNA gene sequence of the strain LN [32]. The resultant PCR product was then sequenced by an automatic sequencing service provided by Mission Biotech Inc. (Taipei, Taiwan). The nucleotide sequence from position 54 to 510 of the 16S rRNA gene sequence of the strain LN, which includes the hypervariable regions V1 to V3, was compared with those from the other type strains of *Bacillus* species. Sequences were aligned in the BioEdit Sequence Alignment Editor program [33], and a phylogenetic tree was constructed by the neighbor-joining method in ClustalW [34], and displayed with the TreeView program [35].

### ZEN uptake by the strain LN in LB broth

ZEN uptake by the strain LN in LB broth was performed using the methods described by Yi et al. [20]. First, 50 mL of LB broth containing 3.5 ppm of ZEN was inoculated with 1% (v/v) of an overnight culture of the strain LN or *B*. *amyloliquefaciens* ATCC 23350, and incubated at 37°C in an orbital shaker at 250 rpm for 48 h. During the incubation period, aliquots of 1.0 mL were taken at 0, 4, 8, 12, 24, 36, and 48 h to estimate the stage of the cultured cell population by measuring turbidity at 600 nm (OD_600_). After measuring turbidity, the aliquots were centrifuged at 17,000 ×*g* for 10 min at 4°C, and the resultant supernatants were analyzed for ZEN concentration by using high-performance liquid chromatography (HPLC).

### ZEN uptake by the strain LN in phosphate-buffered saline (PBS) buffer

A 5-mL aliquot of the overnight cultures of the strains LN and ATCC 23350 was centrifuged at 9,000×*g* for 10 min at 4°C, and the pellets were washed twice with sterile PBS (0.1 M, pH 7.0). Then, the LN cells or ATCC 23350 cells were added to 5 mL of PBS (0.1 M, pH 7.0) containing 5 ppm of ZEN, to yield a final bacterial concentration of 1×10^10^ colony forming units (CFU) mL^-1^. The mixtures were incubated at 37°C for 24 h on an orbital shaker at 120 rpm. During the incubation period, 1.0-mL aliquots were taken at 0, 4, 8, 12, 24, 36, and 48 h, and centrifuged at 17,000 ×*g* for 10 min at 4°C to harvest the cell pellets. The resultant supernatants were analyzed for ZEN concentration. The collected cells were resuspended in 1 mL of 0.1 M of phosphate-buffered saline (PBS; pH 7.4), sonicated for 10 min with an ultrasonicator (Model XL, Misonix, Farmingdale, NY), and fractioned into intracellular supernatant and cell-wall pellet fractions by subsequent centrifugation at 17,000×*g* for 10 min at 4°C. The cell-wall pellet was extracted by acetonitrile-water (84:16, v/v) and centrifuged at 13000×*g* for 20 min at 4°C. The supernatant was analyzed for ZEN concentration.

### ZEN uptake by the strain LN in ZEN-contaminated corn

ZEN-contaminated corn meal was prepared according to the method described by Mateo et al. [36]. In brief, the corn kernels were purchased from local supermarket outlets and tested for the absence of ZEN. They were then autoclaved at 121°C for 15 min. Then, *F*. *graminearum* ATCC 26557 was inoculated in the sterile corn kernels, and incubated at 20°C for 3 weeks. The moldy corn kernels were dried at 45°C for 48 h, and finally ground to meal with a blender (Dynamics Corporation, New Hartford, CT).

To determine the ZEN removal ability of the strain LN in ZEN-contaminated corn, 40 g of the ZEN-contaminated corn meal was suspended in 160 mL of distilled water. After being autoclaved at 121°C for 15 min, the corn meal medium, which contained 1.56 ppm of ZEN, was inoculated with 2 mL of an overnight culture of the strains LN or *B*. *amyloliquefaciens* ATCC 23350, and incubated at 37°C in an orbital shaker at 250 rpm for 48 h. During incubation, samples were taken at 0, 12, 24, 36, and 48 h to extract and quantify the ZEN concentration by using HPLC.

### Determination of ZEN concentration by using HPLC

The ZEN concentration in the samples was determined by using HPLC according to the method described by Yi et al. [20]. Before the HPLC analysis, the samples were extracted with acetonitrile-water (84:16, v/v) for 90 min at 180 rpm on an orbital shaker, cleaned up by using a Romer Mycosep 224 column (Romer Labs Inc.), and evaporated to dryness under nitrogen flow at 60°C according to the manufacturer’s instruction. The dried residue was re-dissolved in 300 μL of methanol solution (80:20, v/v with ultrapure and deionized water) and filtered through a 0.20-μm GHP Acrodisc® syringe filter (Pall Life Sciences, Ann Arbor, MI, USA). Then, 20 μL was injected into the HPLC instrument to quantify ZEN in each sample. The HPLC analysis was performed using a LC-20 AT delivery system (Shimadzu, Kyoto, Japan) equipped with a RF-10AXL fluorescence detector (Shimadzu), a Cosmosil 5C18-ARII column (Nacalai Tesque Inc., Kyoto, Japan; 250×4.6 mm i.d., particle size 5 μm), and a SIL-10A autoinjector (Shimadzu). The mobile phase was a methanol-water-acetonitrile solution (55:35:10, v/v/v) containing ammonium acetate (15 mM), which had been filtered through a membrane (0.45 μm) and degassed for 5 min before use. The mobile phase flow rate was adjusted to 1.0 mL min^-1^, and the detection was performed at 452 nm (excitation) and 271 nm (emission). The linearity of the method was verified by analyzing six standard solutions in the range of 0–8 ppm ZEN, and the ZEN levels in the samples were calculated by comparing the area under the chromatographic peak of the sample with that of the standard solutions.

### Safety assessment of the strain LN

To determine whether the strain LN produced enterotoxin, PCR and enzyme linked immunosorbent assay (ELISA) were used to detect if enterotoxin genes or their products were present in the LN strain. The *B*. *amyloliquefaciens* ATCC 23350, *B*. *cereus* ATCC 11778, and *B*. *cereus* ATCC 33019 purchased from Bioresource Collection and Research Center of Food Industry Research and Development Institute (Hsinchu, Taiwan) were used as the controls.

#### Detection of enterotoxin genes by PCR

The presence of *B*. *cereus* enterotoxin genes *hbl* (A, B, C, and D) and *nhe* (A, B, and C) was determined using PCR as previously described to profile food-poisoning *Bacillus* strains [37,38]. The primers used for amplification of the *hbl* (A, B, C, and D) and *nhe* (A, B, and C) enterotoxin genes are shown in Table 2.

**Table 2:** Primers used for PCR in this study.

Gene	Primer pair	Primer sequence	Fragment size (bp)	Reference
16S rRNA	16S-27f	5’agagtttgatcmtggctcag3’	1,300	[32]
	16S-1492r	5’cggttaccttgttacgactt3’		
*hblA*	HAF	5’ATGATAAAAAAAATCCCTTACAA3’	1,154	[37]
	HAR	5’TTTGTGGAGTAACAGTTTCTACTT3’		
*hblB*	HBF	5’AAGCAATGGAATACAATGGG3’	2,684	[37]
	HBR	5’AATATGTCCCAGTACACCCG3’		
*hblC*	HCF	5’GATACCAATGTGGCAACTGC3’	740	[37]
	HCR	5’TTGAGACTGCTCGCTAGTTG3’		
*hblD*	HDF	5’ACCGGTAACACTATTCATGC3’	829	[37]
	HDR	5’GAGTCCATATGCTTAGATGC3’		
*nheA*	NAF	5’GCTCTATGAACTAGCAGGAAAC3’	755	[37]
	NAR	5’GCTACTTACTTGATCTTCAACG3’		
*nheB*	NBF	5’TTTAGTAGTGGATCTGTACGC3’	743	[37]
	NBR	5’TTAATGTTCGTTAATCCTGC3’		
*nheC*	NCF	5’TGGATTCCAAGATGTAACG3’	683	[37]
	NCR	5’ATTACGACTTCTGCTTGTGC3’		
*bceT*	BTF	5’CGTATCGGTCGTTCACTCGG3’	661	[37]
	BTR	5’GTTGATTTTCCGTAGCCTGGG3’		

#### Detections of enterotoxins by ELISA

The HblC subunit of the Hbl enterotoxin, and the NheA subunit of the Nhe enterotoxin, were detected using the BCET-RPLA Toxin Detection kit (Oxoid, Basingstoke, United Kingdom) and the TECRA Bacillus Diarrheal Enterotoxin Visual Immunoassay (VIA) kit (Tecra Diagnostics, Roseville, Australia), respectively, according to the manufacturer’s instructions.

### Probiotic properties of the strain LN

#### Acidic tolerance of the strain LN

Acid tolerance of the strain LN was determined according to the method described by Liu et al. [39]. A 1-mL aliquot of the overnight culture of LN or ATCC 23350 was centrifuged at 8,000×g for 20 min at 4°C. The pellets were washed twice in sterile PBS (100 mM, pH 7.4), and then resuspended in 10 mL of sterile PBS (100 mM, pH 2.0 or 3.0). The bacterial suspensions were incubated statically at 37°C for 3 h. During the incubation period, a 0.1-mL aliquot of sample was taken at 0, 0.5, 2, and 3 h respectively to count the cell numbers using the standard agar plate method.

#### Bile salt tolerance of the strain LN

Bile salt tolerance of the strain LN was determined as previously described by Liu et al. [39]. Briefly, a 1-mL aliquot of overnight culture of LN or ATCC 23350 was inoculated into 100 mL of LB broth containing 0.3% (w/v) ox gall (Sigma), and incubated at 37°C in an orbital shaker at 250 rpm for 12 h. During the incubation period, a 1-mL aliquot of sample was taken at 0, 4, 8, and 12 h, respectively, to estimate the cell population by measuring turbidity at OD_600_.

#### Anti-pathogenic activities of the strain LN

The pathogens used for the evaluation of anti-pathogenic activity of the strain LN included *Bacillus cereus* ATCC 11778, *Bacillus cereus* ATCC 33019, *Escherichia coli* O157:H7 ATCC 35150, *Listeria monocytogenes* BCRC 14930, *Listeria monocytogenes* BCRC 15338, *Listeria monocytogenes* BCRC 15387, and *Salmonella enterica* subsp. *enterica* ATCC 9184. All the pathogens were purchased from the Bioresource Collection and Research Center of Food Industry Research and Development Institute (Hsinchu, Taiwan).

A 5-mL aliquot of the overnight culture of the strains LN and ATCC 23350 was centrifuged at 9,000×*g* for 10 min at 4°C. The resultant supernatants were adjusted to pH 7.0, and then were sterilized by filtering through a 0.22-μm nylon filter. Then, 50 μL of the filtered neutralized supernatants were added to filter paper discs (6 mm diameter), which were placed on the agar plate surface previously inoculated with indicator pathogens. The agar was incubated at 37°C for 24 h. After incubation, the formation of clear zone around the discs indicated a positive anti-pathogenic activity of the metabolites on the pathogens; thus, the diameter of a clear zone around the discs was measured.

### Statistical analysis

All results were analyzed using the general linear-model procedure available with the Statistical Analysis System software package version 9.1 (Statistical Analysis System Institute 2002). Duncan’s multiple range test [40] was used to detect the differences between treatment means. All results were expressed as mean ± standard deviation, and a *P*-value less than 0.05 was considered significant.

## Results

### Isolation and identification of *Bacillus* sp. LN

**Fig 1:**
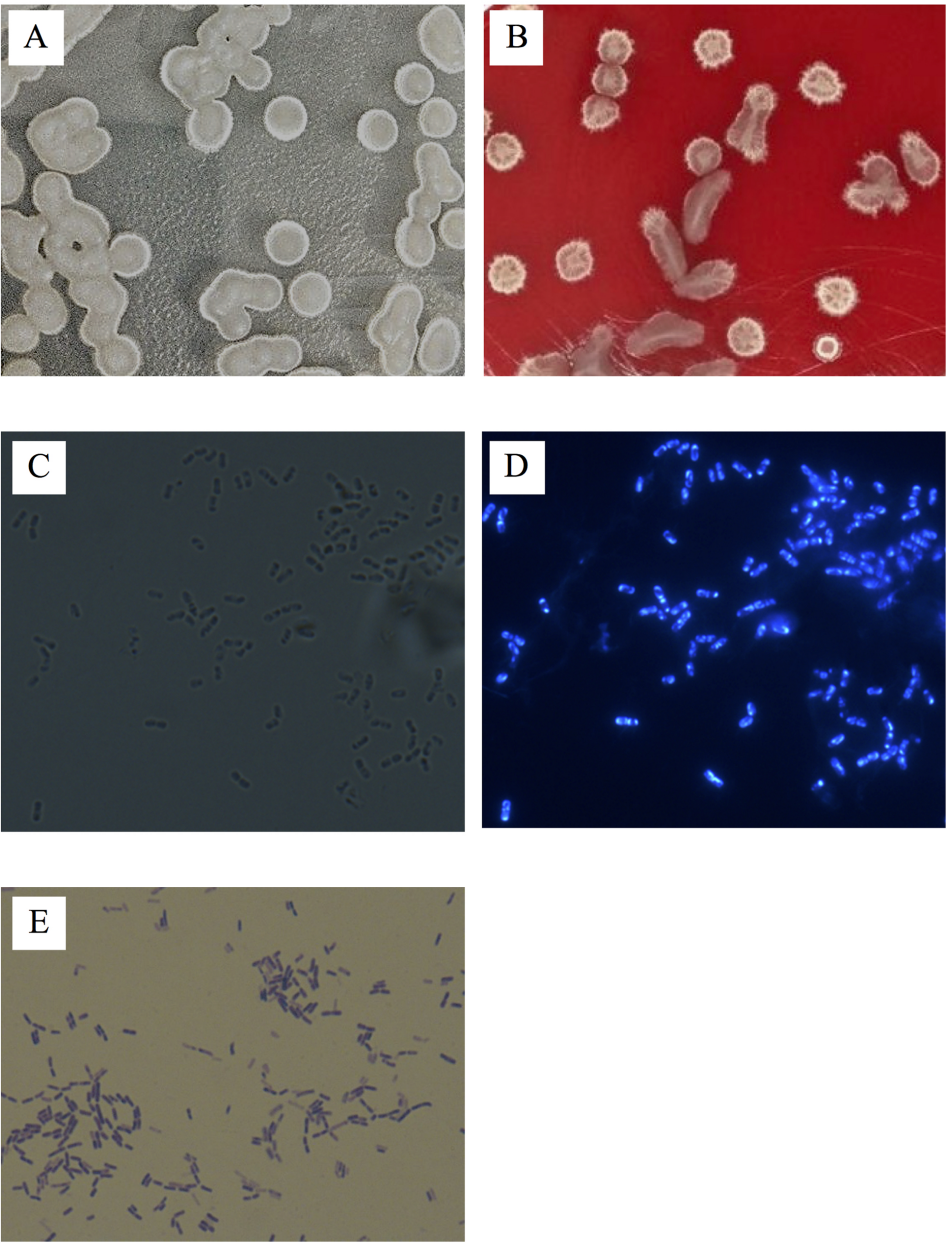
Macroscopic examination of *Bacillus amyloliquefaciens* LN. (A) and (B) The colony morphologies of LN on LB agar and blood agar plates, respectively. (C) and (D) The LN cells observed under phase-contrast and fluorescence microscopes, respectively. (E) Gram-stained LN cells observed under a phase-contrast microscope.

A total of 148 bacterial strains were isolated from the moldy corn samples, and each bacterial isolate was evaluated for ZEN removal abilities. Among these isolates, a pure culture of strain LN showed the greatest ability to remove ZEN. Further analysis on the basis of phenotypic (including catalase and oxidase reactions and typical spore formation), and physiological characteristics (including anaerobic growth, growth at 50 and 55°C, and assimilation of citrate, nitrate, and propionate) indicated that the strain LN was closely related to genus *Bacillus*. Macroscopic observation showed that the strain LN exhibited a smooth and sticky surface, protruding shape, and irregular edge colony morphology, and did not cause hemolysis on the blood agar plate (Fig 1A and 1B). Microscopic observation showed the cells of the strain LN appeared as straight rods with rounded ends, arranged singly or in chains, and were motile and endospore-forming (Fig 1C and 1D). The strain LN cells appeared purple after Gram staining (Fig 1E), indicating the strain LN was Gram positive. The features of the strain LN were consistent with the description of *Bacillus amyloliquefaciens* in Bergey’s Manual of Systematic Bacteriology [41].

**Fig 2:**
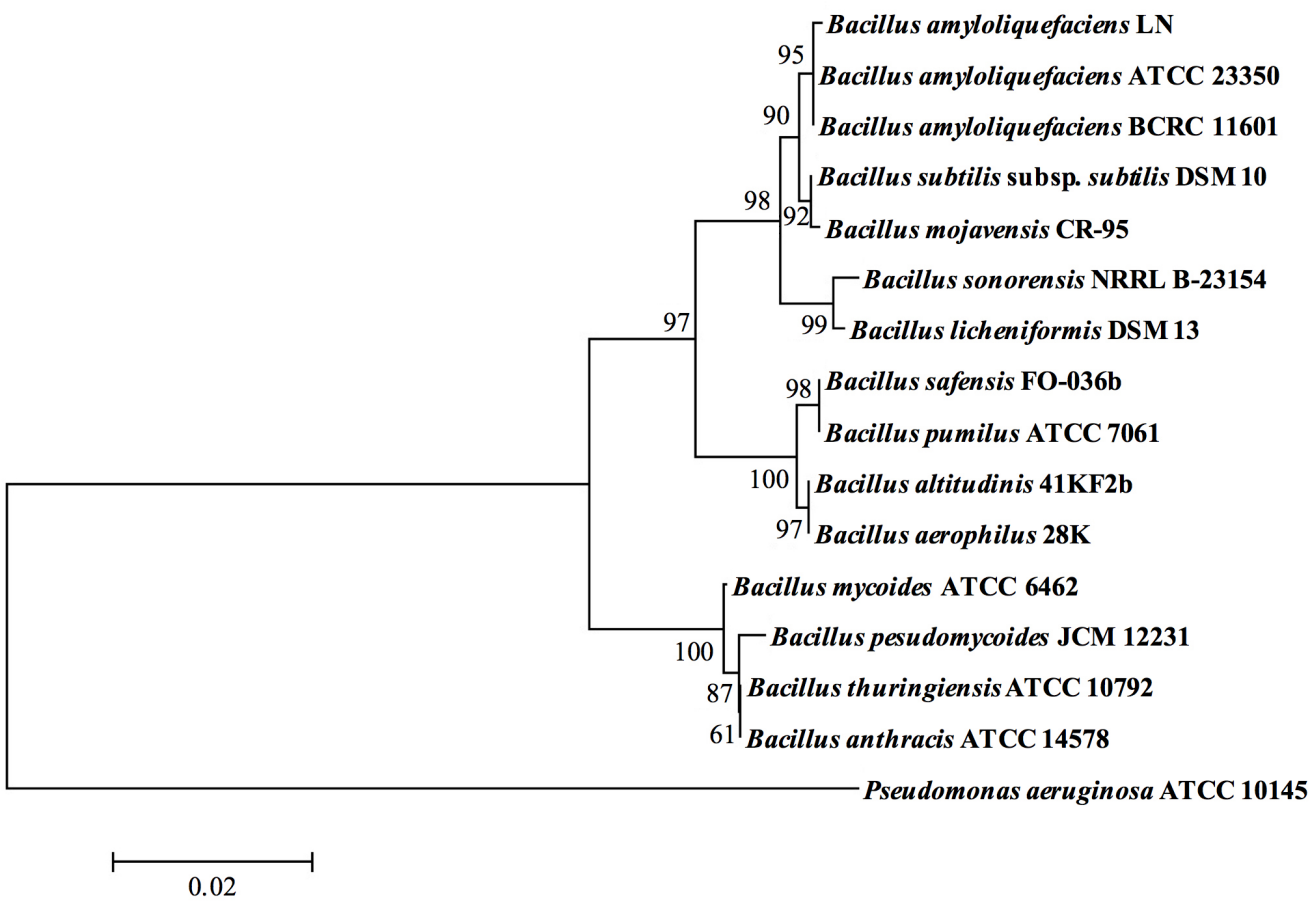
Neighbor-joining phylogenetic tree-based study of the V1-V3 region of the 16S rRNA gene of *Bacillus* species. At major nodes, bootstrap percentages for 1,000 re-samplings are shown. The scale bar represents 0.1 nucleotide substitution per nucleotide position. *Bacillus* species include *Bacillus aerophilus* 28K (GenBank accession no. AJ831844), *B*. *altitudinis* 41KF2b (AJ831842), *B*. *amyloliquefaciens* BCRC 11601 (NR_116022), *B*. *amyloliquefaciens* ATCC 23350 (X60605), *B*. *amyloliquefaciens* LN (KP261025), *B*. *anthracis* ATCC 14578 (KC119183), *B*. *licheniformis* DSM 13 (X68416), *B*. *mojavensis* CR-95 (AY603656), *B*. *mycoides* ATCC 6462 (NR_115993), *B*. *pseudomycoides* JCM 12231 (LC107614), *B*. *pumilus* ATCC 7061 (AY876289), *B*. *safensis* FO-036b (AF234854), *B*. *sonorensis* NRRL B-23154 (NR_025130), *B*. *subtilis* subsp. *subtilis* DSM 10 (AJ276351), and *Bacillus thuringiensis* ATCC 10792 (NR_114581). *Pseudomonas aeruginosa* ATCC 10145 (NR_114471) was used as an outgroup to root the tree.

The biochemical characteristics of the strain LN were compared with that of *B*. *amyloliquefaciens* ATCC 23350 by using the API 50 CHB system. Analysis of carbon source utilization profiles indicated that both strains LN and *B*. *amyloliquefaciens* ATCC 23350 grew on 21 out of 49 carbohydrates. However, distinct variation was observed in the utilization of the sugars N-acetylglucosamine, dulcitol, D-galactose, D-glucose, D-lactose, D-mannose, melibiose, and D-xylose (Table 3). Based on carbon source utilization, the strain LN was identified as *B*. *amyloliquefaciens* with 99.8% certainty. To further confirm the identification of the strain LN with the species *B*. *amyloliquefaciens*, the partial sequence for the 16S rRNA gene was included in the phylogenetic analysis. The phylogenetic tree based on the hypervariable regions V1 to V3 of 16S rRNA gene showed that the strain LN exhibited 99.3% identity with *B*. *amyloliquefaciens* ATCC 23350 (Fig 2). Thus, based on the results of microscopic observations, biochemical characteristics, and the phylogenetic analysis based on the V1-V3 region of the 16S rRNA gene, the strain LN was identified to belong to *B*. *amyloliquefaciens*.

**Table 3:** The biochemical characteristics of *Bacillus amyloliquefaciens* LN and *B*. *amyloliquefaciens* ATCC 23350.

Substrate	Strain		Substrate	Strain	
	LN	ATCC23350		LN	ATCC23350
Glycerol	+	+	Esculin ferric citrate	+	+
Erythritol	-	-	Salicin	+	+
D-Arabinose	-	-	D-Cellobiose	+	+
L- Arabinose	+	+	D-Maltose	+	+
D-Ribose	+	+	D-Lactose	+	-
D-Xylose	+	-	Melibiose	-	+
L-Xylose	-	-	D-Sucrose	+	+
D-Adonitol	-	-	D-Trehalose	+	+
Methyl-xylopyranoside	-	-	Inulin	+	+
D-Galactose	-	+	D-Melezitose	-	-
D-Glucose	+	-	D-Raffinose	+	+
D-Fructose	+	+	Amidon/ Starch	+	+
D-Mannose	+	-	Glycogen	+	+
D-Sorbose	+	+	Xylitol	-	-
Rhamnose	-	-	Gentiobiose	-	-
Dulcitol	-	+	D-Turanose	-	-
Inositol	-	-	D-Lyxose	-	-
D-Mannitol	+	+	D-Tagatose	+	+
D- Sorbitol	+	+	D-Frucose	-	-
α-Methyl-D- mannoside	-	-	L- Frucose	-	-
α-Methyl-D- glucoside	+	+	D- Arabitol	-	-
N-Acetylglucosamine	+	-	L-Arabitol	-	-
Amygdalin	+	+	Gluconate	-	-
Arbutin	+	+	2-Ketogluconate	-	-
			5-Ketogluconate	-	-

### Removal of ZEN by *B*. *amyloliquefaciens* LN

**Fig 3:**
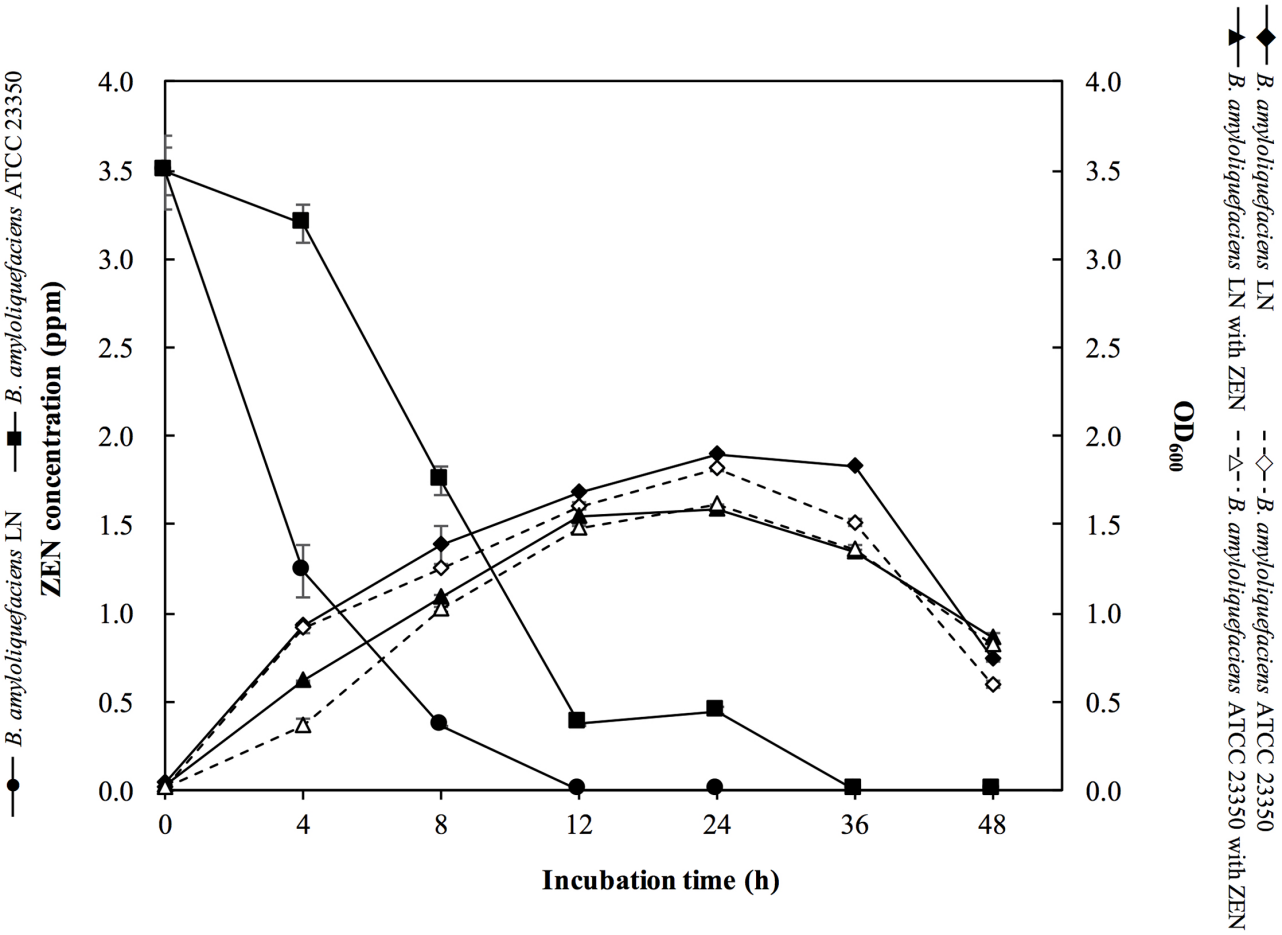
Growth curves *Bacillus amyloliquefaciens* LN and *B*. *amyloliquefaciens* ATCC 23350 cultured in LB broth with or without ZEN (3.5 ppm), and ZEN degradation kinetics of *B*. *amyloliquefaciens* LN and *B*. *amyloliquefaciens* ATCC 23350 in LB broth with ZEN (3.5 ppm). The bars represent standard errors of the means.

The ZEN removal ability of *B*. *amyloliquefaciens* LN was determined in LB broth containing 3.5 ppm of ZEN. As a reference, and to compare degrading activities, the type strain of *B*. *amyloliquefaciens* ATCC 23350, was cultured and investigated using the same procedure. As shown in Fig 3, *B*. *amyloliquefaciens* LN and *B*. *amyloliquefaciens* ATCC 23350 reached the highest cell density (OD_600_) of 1.90 and 1.68, respectively, when cultured in LB broth without ZEN for 8 h. *B*. *amyloliquefaciens* LN and *B*. *amyloliquefaciens* ATCC 23350 also reached the highest cell density of 1.81 and 1.62, respectively, when cultured in LB broth containing 3.5 ppm of ZEN for 8 h. Both *B*. *amyloliquefaciens* LN and *B*. *amyloliquefaciens* ATCC 23350 displayed characteristic ZEN removal abilities. *B*. *amyloliquefaciens* LN removed almost all of ZEN in the LB broth after 24 h of incubation, while *B*. *amyloliquefaciens* ATCC 23350 removed almost all of ZEN in the LB broth after 36 h of incubation (Fig 3). These results indicated that *B*. *amyloliquefaciens* LN and *B*. *amyloliquefaciens* ATCC 23350 grew well in LB containing 3.5 ppm of ZEN, and showed ZEN removal abilities.

**Fig 4:**
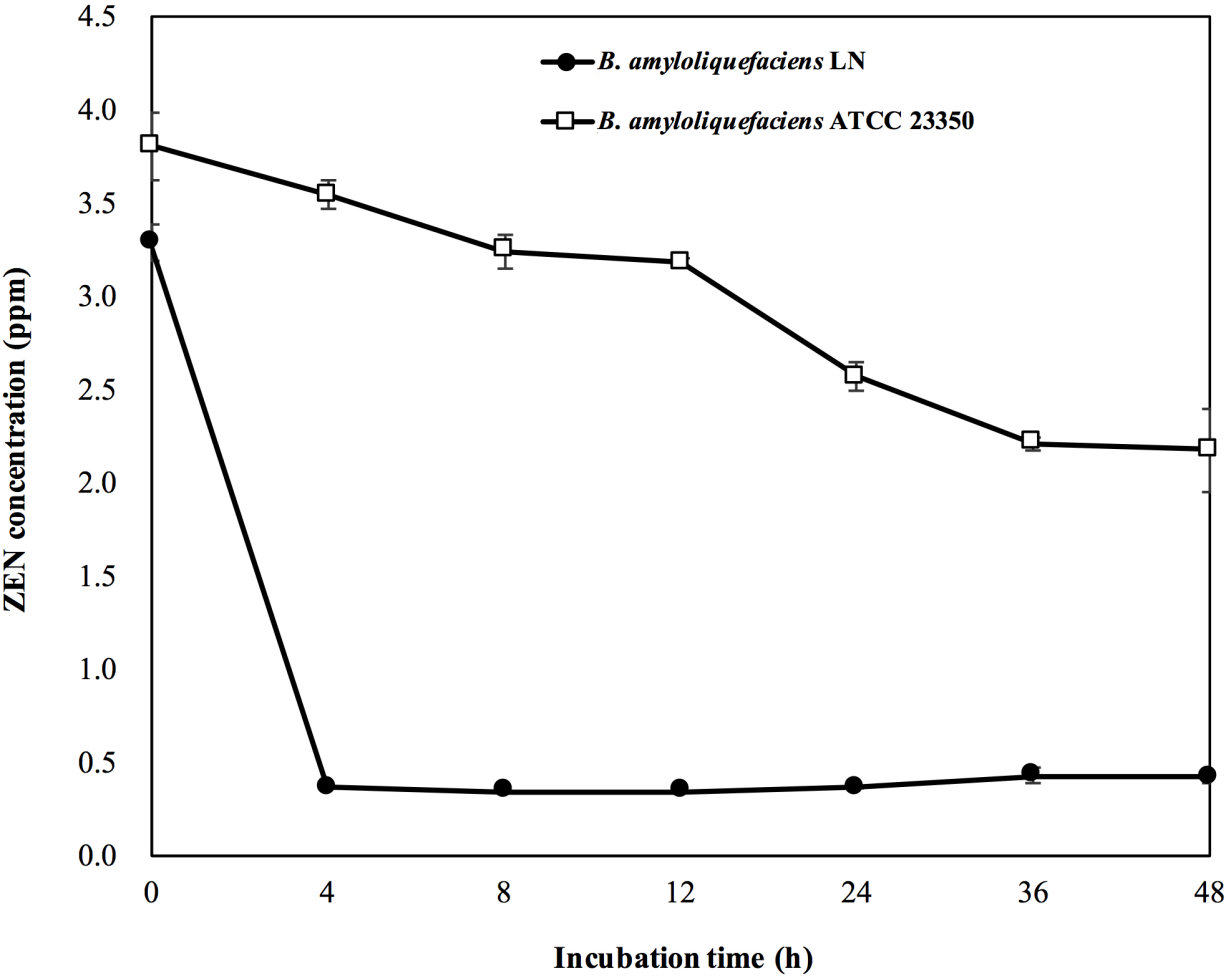
ZEN degradation kinetics of *Bacillus amyloliquefaciens* LN and *B*. *amyloliquefaciens* ATCC 23350 in phosphate-buffered saline (PBS; 0.1 M, pH 7.0) containing 5 ppm of ZEN.

The ZEN removal abilities of *B*. *amyloliquefaciens* LN and ATCC 23350 were also tested in the PBS containing 5 ppm of ZEN. Immediately after adding of *B*. *amyloliquefaciens* LN cells to the PBS containing ZEN, the ZEN concentration in the PBS was reduced from 5 ppm to 3.28 ppm, i.e., more than 34.4% of ZEN was adsorbed by *B*. *amyloliquefaciens* LN cells (Fig 4). On the other hand, immediately after adding of *B*. *amyloliquefaciens* ATCC 23350 cells to the PBS containing ZEN, the ZEN concentration in the PBS was reduced from 5 ppm to 3.80 ppm, i.e., 24.0% of ZEN was adsorbed by *B*. *amyloliquefaciens* ATCC 23350 cells (Fig 4). These results indicated that the ZEN adsorption ability of *B*. *amyloliquefaciens* LN was better than that of *B*. *amyloliquefaciens* ATCC 23350. After incubation of *B*. *amyloliquefaciens* LN in the PBS containing 5 ppm of ZEN for 4 h, the ZEN concentration in the PBS was reduced to 0.36 ppm, i.e., more than 92.8% of ZEN was removed by *B*. *amyloliquefaciens* LN. In addition, the amounts of ZEN recovered from the cells after 4 h of incubation was almost the same as that recovered from the *B*. *amyloliquefaciens* LN cells immediately after coming into contact with ZEN. Since the amounts of ZEN adsorbed by the cells of *B*. *amyloliquefaciens* LN cells did not increase with the extension of incubation time, but the amounts of residual ZEN in PBS decreased with the extension of incubation time, we suggested that *B*. *amyloliquefaciens* LN appears to possess ZEN degrading ability. On the other hand, *B*. *amyloliquefaciens* ATCC 23350 decreased the ZEN concentration from 5 ppm to 2.17 ppm after 48 h of incubation, i.e., only about 56.6% of ZEN was removed by *B*. *amyloliquefaciens* ATCC 23350 (Fig 4). In addition, the amounts of ZEN recovered from the *B*. *amyloliquefaciens* ATCC 23350 cells after 48 h of incubation was almost the same as that recovered from the cells immediately after coming into contact with ZEN. This phenomenon was similar to that observed for *B*. *amyloliquefaciens* LN cells, indicating that *B*. *amyloliquefaciens* ATCC 23350 also possessed ZEN degrading ability. However, the ZEN removal ability of *B*. *amyloliquefaciens* ATCC 23350 was lower than that of *B*. *amyloliquefaciens* LN.

**Fig 5:**
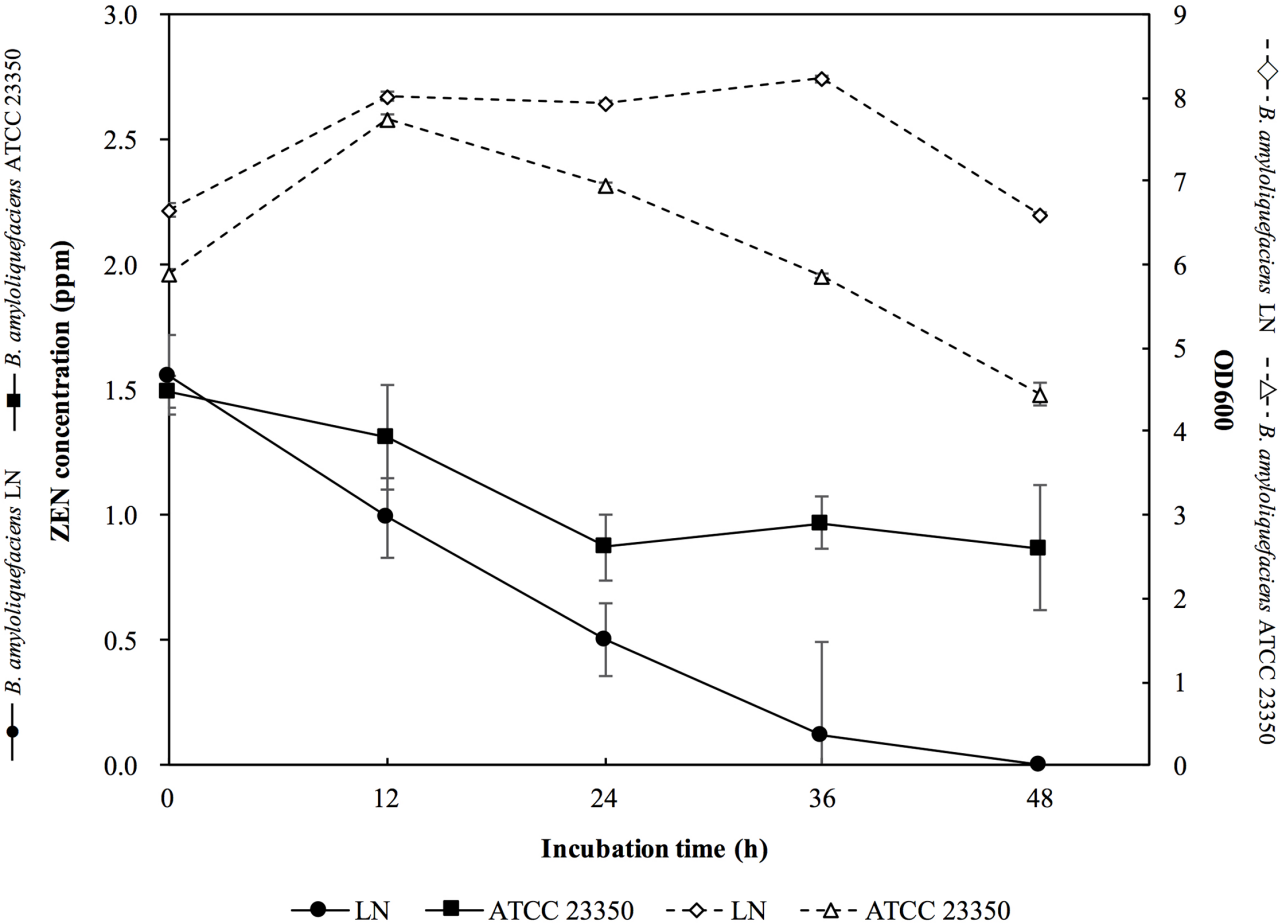
Growth curves and ZEN degradation kinetics of *Bacillus amyloliquefaciens* LN and *B*. *amyloliquefaciens* ATCC 23350 in corn meal medium containing 1.56 ppm ZEN. The bars represent standard errors of the means.

The ZEN removal abilities of *B*. *amyloliquefaciens* LN and ATCC 23350 were further confirmed in ZEN-contaminated corn. Before inoculation of the tested bacterial strains, the ZEN concentration in the corn meal medium was 1.56 ppm. Approximately 6.66 log CFU/mL of bacteria were inoculated into the corn meal medium. After 36 h of incubation, the cell number of *B*. *amyloliquefaciens* LN increased to 8.23 log CFU mL^-1^ while that of *B*. *amyloliquefaciens* ATCC 23350 was 6.03 log CFU mL^-1^ (Fig 5), indicating that the growth of *B*. *amyloliquefaciens* LN in ZEN-contaminated corn meal medium was better than that of *B*. *amyloliquefaciens* ATCC 23350. After 36 h of incubation, ZEN residues in the supernatant of the corn meal medium were 0.12 and 0.97 ppm, respectively, i.e., *B*. *amyloliquefaciens* LN decreased the concentration of ZEN 92.3%, while *B*. *amyloliquefaciens* ATCC 23350 decreased the concentration of ZEN 37.8% in the ZEN-contaminated corn meal medium (Fig 5). These results indicated that the ZEN removal ability of *B*. *amyloliquefaciens* LN was significantly greater than that of *B*. *amyloliquefaciens* ATCC 23350.

### Safety assessment of *B*. *amyloliquefaciens* LN

To confirm the safety of *B*. *amyloliquefaciens* LN, PCR was used to detect whether the enterotoxin genes were present in the genome, as described previously for profiling of food-poisoning *Bacillus* strains [37,38]. The type strain of *B*. *amyloliquefaciens*, ATCC 23350, and the two enterotoxin-producing strains, *B*. *cereus* ATCC 11778 and ATCC 33019, were also tested using the same procedure. The results revealed that neither *B*. *amyloliquefaciens* strains carry the *B*. *cereus* enterotoxin genes *hbl* (A, B, C, and D) or *nhe* (A, B, and C) (Table 4). *B*. *amyloliquefaciens* LN was further examined with two commercial immunoassay kits specific for the HblC subunit of the Hbl enterotoxin, and the NheA subunit of the Nhe enterotoxin, to determine whether *B*. *amyloliquefaciens* LN produced enterotoxins. The results demonstrated that neither *B*. *amyloliquefaciens* LN nor *B*. *amyloliquefaciens* ATCC 23350 produced the enterotoxins tested for (Table 4).

**Table 4:** Results of enterotoxin detection.

Strain	Hbl enterotoxin ^a^				Nhe enterotoxin ^a^			BceT enterotoxin	Tecra kit test index ^b^
	*hblA*	*hblB*	*hblC*	*hblD*	*nheA*	*nheB*	*nheC*	*bceT1*	
*Bacillus amyloliquefaciens* LN	-	-	-	-	-	-	-	-	1
*Bacillus amyloliquefaciens* ATCC 23350	-	-	-	-	-	-	-	-	1
*Bacillus cereus* ATCC 11778	-	-	-	-	+	+	+	+	4
*Bacillus*. *cereus* ATCC 33019	+	+	+	+	+	+	+	+	4

^a^+: PCR positive; -: PCR negative.

^**b**^ According to the manual of the kit, score less than 3 represents negative.

### Probiotic properties of *B*. *amyloliquefaciens* LN

**Fig 6:**
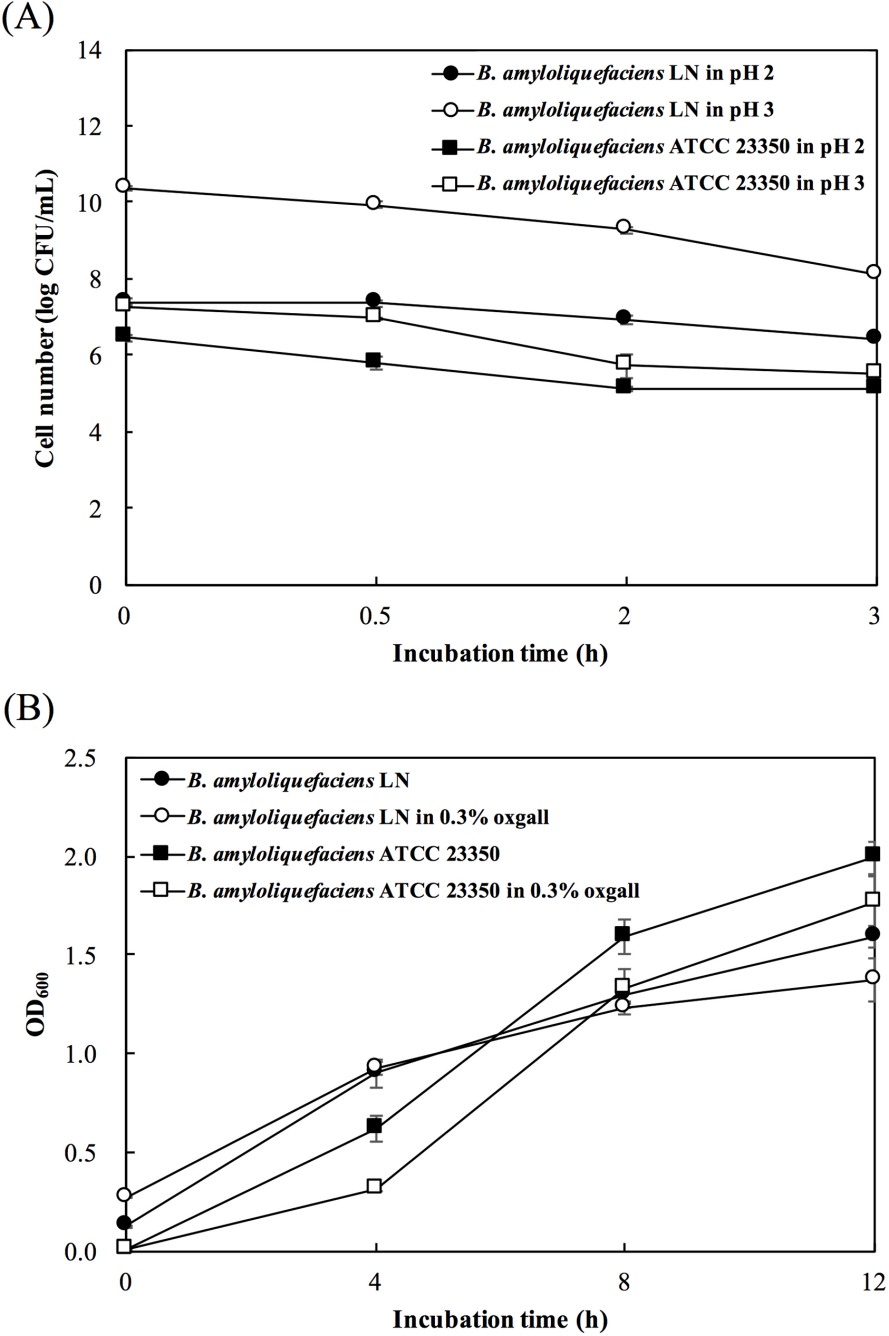
**Survival of *Bacillus amyloliquefaciens* LN and *B*. *amyloliquefaciens* ATCC 23350 after incubation at pH 2.0 or 3.0 (A) or in the presence of 0.3% oxgall (B).** The bars represent standard errors of the means.

*B*. *amyloliquefaciens* LN was further examined for the probiotic properties, including acidic tolerance, bile salt tolerance, and anti-pathogenic activities. The acid tolerance of *B*. *amyloliquefaciens* strains was evaluated by culturing the bacterial cells in PBS at pH 2.0 or 3.0 for 3 h, and the bile-salt tolerance of the *B*. *amyloliquefaciens* strains was determined by culturing the bacterial cells in LB broth containing 0.3% oxgall for 12 h. As shown in Fig 6, both *B*. *amyloliquefaciens* strains survived after incubation at pH 2.0 or 3.0 for 3 h (Fig 6A), and grew well in LB broth containing 0.3% oxgall (Fig 6B). After incubation in the acidic conditions for 3 h, the bacterial counts of *B*. *amyloliquefaciens* LN were significantly higher than those of *B*. *amyloliquefaciens*, ATCC 23350. After cultured in LB broth containing 0.3% oxgall for 12 h, the bacterial counts of *B*. *amyloliquefaciens* LN were not significantly different from that cultured in LB broth without oxgall. These results indicated that *B*. *amyloliquefaciens* LN has the ability to tolerate acid and bile salts.

The pathogens used for the evaluation of the anti-pathogenic activity of *B*. *amyloliquefaciens* LN included *B*. *cereus* ATCC 11778, *B*. *cereus* ATCC 33019, *E*. *coli* O157:H7 ATCC 35150, *L*. *monocytogenes* BCRC 14930, *L*. *monocytogenes* BCRC 15338, *L*. *monocytogenes* BCRC 15387, and *S*. *enterica* subsp. *enterica* ATCC 9184. As shown in Table 5, *B*. *amyloliquefaciens* LN inhibited the growth of *B*. *cereus* ATCC 11778, *B*. *cereus* ATCC 33019, and *L*. *monocytogenes* BCRC 15338; in contrast, *B*. *amyloliquefaciens* ATCC 23350 did not show any inhibition effect on the tested pathogens.

**Table 5:** Anti-pathogen activities of *Bacillus amyloliquefaciens* LN and *B*. *amyloliquefaciens* ATCC 23350.

Indicator strain	Anti-pathogen activity (Inhibition zone in mm)	
	LN	ATCC 23350
*Bacillus cereus* ATCC 11778	9.96 ± 0.14^a^	NI^b^
*Bacillus cereus* ATCC 33019	8.64 ± 0.24	NI
*Escherichia coli* O157:H7 ATCC 35150	NI	NI
*Listeria monocytogenes* BCRC 14930	NI	NI
*Listeria monocytogenes* BCRC 15338	10.63 ± 0.59	NI
*Listeria monocytogenes* BCRC 15387	NI	NI
*Salmonella enterica* subsp. *enterica* BCRC 12947	NI	NI

^a^ Values are the mean ± standard deviations of triplicate measurements.

^b^ NI: no inhibition.

## Discussion

There are several strategies available for decontamination of ZEN in foods and feeds, and these include chemical methods such as exposure of ZEN-containing foods to acids, bases, ozone, or hydrogen peroxide; physical methods such as thermal inactivation and inactivation through irradiation; and biological methods such as using adsorbing agents to decrease the bioavailability of ZEN or using biotransforming agents to degrade ZEN into non-toxic metabolites [4,11]. Among the ZEN decontamination methods, biological methods are preferable because they provides the opportunity for removal of ZEN under mild conditions, without the use of harmful chemicals, or causing significant losses in nutritive value and palatability of decontaminated food and feed [11,20]. In this study, we found that *B*. *amyloliquefaciens* LN reduced the ZEN concentration in PBS from 5 ppm to 3.28 ppm immediately after coming into contact with ZEN (Fig 4), indicating that *B*. *amyloliquefaciens* LN cells may possess ZEN adsorption ability. We also found that *B*. *amyloliquefaciens* LN further reduced the ZEN concentration in PBS to 0.36 ppm after 4 h of incubation, and the amount of ZEN adsorbed by the cells of *B*. *amyloliquefaciens* LN cells did not increase with the extension of incubation time. These results indicated that *B*. *amyloliquefaciens* LN possess ZEN removal ability.

The most commonly used biological mycotoxin-adsorbing agents are yeast cell walls derived from *Saccharomyces cerevisiae*. In addition to yeast cell walls, some lactic acid bacterial strains such as *L*. *rhamnosus* GG and *L*. *rhamnosus* LC-705 are also used because of their wider range of mycotoxin adsorption abilities [42]. It is known that physical adsorption, which usually occurs at low temperatures in a rapid fashion, plays an important role in the mycotoxin adsorption reaction of biological mycotoxin-adsorbing agents [43]. The observation that *B*. *amyloliquefaciens* LN reduced the ZEN concentration from 5 ppm to 3.28 ppm immediately after coming into contact with ZEN suggests that the *B*. *amyloliquefaciens* LN cells are able to adsorb ZEN. The mycotoxin adsorption abilities of Gram-positive bacteria are mainly attributed to cell wall components; for example, the peptidoglycans extracted from *B*. *subtilis* showed a fumonisin B1 adsorption ability [44], while the polysaccharide components of the cell wall of *L*. *rhamnosus* possess ZEN adsorption ability [12]. The adsorption abilities of the biological mycotoxin-adsorbing agents are affected by heat treatments, because heat treatments reduce the thickness of the peptidoglycan, increase the pore size of peptidoglycan structure, and break the glycosidic linkages of the polysaccharide [12,13,45,46]. The adsorption abilities of the biological mycotoxin-adsorbing agents are also affected by acid treatments, because the acid reduces the thickness of the peptidoglycan, increases the pore size of the peptidoglycan structure, and breaks the amide linkages in the structure of peptidoglycans [12,13,45,46]. Therefore, the ZEN adsorption ability of biological mycotoxin-adsorbing agents may be changed when they enter the acidic gastric environment, or are exposed to heat during the pelleting process. For this reason, future research will be directed towards identifying the cell components responsible for the adsorption of ZEN by *B*. *amyloliquefaciens* LN, and determining the effects of acid and heat treatments on the ZEN adsorption ability of *B*. *amyloliquefaciens* LN.

In addition to the use of biological mycotoxin-adsorbing agents, biotransforming agents are used for decontamination of ZEN in feedstuffs or food ingredients. Biotransforming agents, including *B*. *subtilis* [19], *B*. *amyloliquefaciens* ZDS-1[29], *B*. *licheniformis* CK1 [20], *Pseudomonas fluorescens* MM1 [14], *P*. *putida* ZEA-1 [47], and *Rhodococcus pyridinivorans* K408 [48] convert ZEN into less- or non-toxic metabolites. Though many bacterial strains possess ZEN degrading activity, their applications for the detoxification of food and feed has been limited. This may be due to insufficient scientific or clinical evidence for the safety of the microorganisms towards animals [49]. *B*. *amyloliquefaciens* has been used for decades to produce a variety of enzymes, including α-amylase, cellulase, and protease for food processing [26]. In 1999, the United States Food and Drug Administration published a final rule in the Federal Register affirming that carbohydrase and protease enzyme preparations from *B*. *amyloliquefaciens* are generally recognized as safe (GRAS) for use as direct food ingredients [50]. However, some strains of *B*. *subtilis*, *B*. *licheniformis*, *B*. *circulans*, *B*. *megaterium*, *B*. *lentimorbis*, *B*. *amyloliquefaciens*, and *B*. *lentus* are reported to contain enterotoxin genes or to produce enterotoxins [51]. Most food poisoning incidents attributed to *Bacillus* species are associated with *B*. *cereus*, and it is believed that the enterotoxins produced by *Bacillus* spp. other than *B*. *cereus* are proteins transcribed from genes that are similar to those of *B*. *cereus* enterotoxins [51]. In the present study, we demonstrated that *B*. *amyloliquefaciens* LN did not carry the *B*. *cereus* enterotoxin genes *hbl* (A, B, C, and D) and *nhe* (A, B, and C), did not produce the enterotoxin protein subunits HblC and NheA (Table 4), and did not cause hemolysis on the blood agar plate (Fig 1B). Future research will be conducted to evaluate the safety of *B*. *amyloliquefaciens* LN for use in animal feed following the methods described by Pariza et al. [52].

To evaluate the probiotic potential, the acidic and bile salt tolerance of *B*. *amyloliquefaciens* LN was determine. A probiotic potential strain must be able to resist to the digestion process in the gastrointestinal tract [53]. In the acidic conditions in the stomach, and the bile salts in the duodenum, are two of the most difficult hurdles associated with the survival of the probiotics. Gastric acid in the stomach represents a primary defense mechanism against the majority of ingested microorganisms, while bile salts in duodenum reduces survival of bacteria due to the fact that lipids and fatty acids, which comprise the bacterial cell membranes, are highly susceptible to the destruction by bile salts [54]. In this study, *B*. *amyloliquefaciens* LN survived after incubation at pH 2.0 or 3.0 for 3 h (Fig 6A) or in LB broth containing 0.3% oxgall for 12 h (Fig 6B). These results indicated that *B*. *amyloliquefaciens* LN might be able to survive transit through the stomach, and survive in the intestinal environment where it can work effectively.

Another important property of probiotic potential strains is their antimicrobial activity against potentially pathogenic bacteria [55]. As shown in Table 5, *B*. *amyloliquefaciens* LN inhibited the growth of *B*. *cereus* ATCC 11778, *B*. *cereus* ATCC 33019, and *L*. *monocytogenes* BCRC 15338. *B*. *cereus* and *L*. *monocytogenes* are well known foodborne pathogens that cause a wide range of opportunistic infections of humans and animals [56,57]. *B*. *cereus* is easily spread from its natural environment to foods and feeds because its endospores are resistant to various stresses, and exhibit the capacity for long-term survival [58]. Data from the late 20th century suggest that diarrheal syndrome caused by *B*. *cereus* was reported as the most frequent isolate from foodborne illness in 1990 in Norway, and was a common occurrence in Finland, Hungary, Taiwan, and the Netherlands [57]. *L*. *monocytogenes* is another important foodborne pathogen, which causes listeriosis in humans and animals. It is estimated that *L*. *monocytogenes* causes an estimated 1,591 cases of illness, 1,455 hospitalizations, and 255 deaths annually in the United States [59]. Live animals are the main source of contamination, and may transmit *L*. *monocytogenes* to humans. Therefore, a reduction of the intestinal carrier rate of *L*. *monocytogenes* in pigs at the herd level could reduce contamination at the slaughterhouse [56]. Several bacteriocin-like substances, such as surfactin, iturin, fengycin, and bacillopeptins, produced by *Bacillus* spp. have been reported to possess antagonistic activity against pathogenic bacteria, including *B*. *cereus* and *L*. *monocytogenes* [60,61]. In the present study, we found that *B*. *amyloliquefaciens* LN inhibited the growth of both *B*. *cereus* strains tested in this study, which suggested that *B*. *amyloliquefaciens* LN has the potential to be used for prevention and control of *B*. *cereus*. We also found that *B*. *amyloliquefaciens* LN inhibited the growth of *L*. *monocytogenes* BCRC 15338 but did not inhibit the growth of *L*. *monocytogenes* BCRC 14930 and *L*. *monocytogenes* BCRC 15387. It has been demonstrated that *L*. *monocytogenes* has a high degree of genetic diversity at the species level [59], resulting in the wide diversity of bacteriocin resistance among *L*. *monocytogenes* strains [60]. Future research will be directed towards identifying the anti-pathogenic agents produced by *B*. *amyloliquefaciens* LN, and determining their potential application in preventing listeriosis.

Recently, some *B*. *amyloliquefaciens* strains were reported to have aflatoxins, ochratoxin, or ZEN degrading ability [27–29]. Siahmoshteh et al. reported that *B*. *amyloliquefaciens* UTB2 could inhibit *Aspergillus* growth and degrade aflatoxin B1 [27]. Chang et al. found that *B*. *amyloliquefaciens* ASAG1 displayed an ochratoxin degrading ability due to its carboxypeptidase activity [28]. Xu et al. reported that *B*. *amyloliquefaciens* ZDS-1 showed an efficient ZEN degrading activity, which decreased 95.7% of ZEN in culture medium containing 3 ppm of ZEN and degraded 62.1% of ZEN in wheat contaminated with 1 ppm of ZEN [29]. Despite the many publications of biodegradation of mycotoxins by *B*. *amyloliquefaciens* strains, their application in practice in detoxification of food and feed has been limited. This may be due to insufficient scientific evidence for the safety and probiotic properties of these mycotoxin-degrading *B*. *amyloliquefaciens* strains. In the present study, we found that *B*. *amyloliquefaciens* LN removed almost all of ZEN in LB broth contain 3.5 ppm of ZEN and decreased the concentration of ZEN 92.3% in the ZEN-contaminated corn meal medium. In addition, we demonstrated that *B*. *amyloliquefaciens* LN possessed probiotic properties and did not possess the ability to produce enterotoxins. Therefore, *B*. *amyloliquefaciens* LN could potentially be used to reduce the concentrations of ZEN in feedstuffs safely.

## Conclusions

A ZEN-detoxifying strain of *B*. *amyloliquefaciens* was isolated, and genotypically and phenotypically characterized. *B*. *amyloliquefaciens* LN displayed characteristic ZEN removal ability, did not produce enterotoxins, and showed probiotic characteristics including acidic tolerance, bile salt tolerance, and anti-pathogenic activities. These findings suggest that *B*. *amyloliquefaciens* LN has the potential to be used as a feed additive to reduce the concentrations of ZEN in feedstuffs.
